# Antipsychotics induced constipation in patients with mental disorders. treatment suggestion with prucalopride in refractory cases. case report and literature review

**DOI:** 10.1192/j.eurpsy.2022.2014

**Published:** 2022-09-01

**Authors:** P. Mpouras, P. Argitis, O. Pikou, A. Karampas, S. Karavia, F.-E. Kakavitsas, Z. Chaviaras

**Affiliations:** 1 General Hospital of Corfu, General Medicine, corfu, Greece; 2 General Hospital of Corfu, Psychiatric, Corfu, Greece; 3 General Hospital of Corfu, Dermatology, corfu, Greece

**Keywords:** CONSTIPATION, PRUCALOPRIDE

## Abstract

**Introduction:**

Successful stabilization of patients with mental disorders requires most of the times the use of more than one antipsychotic medications with increase prevalence of clozapine in refractory cases. Constipation consists one of the most debilitating side effect of the therapy, which gradually progresses to a chronic state of bowel movement dysfunction, with recurrent episode of paralytic ileus of various severity.

**Objectives:**

We describe the case of a middle age male treated with clozapine for refractory mental disorder, who developed ileus and subsequent bowel dysfunction not amenable to laxatives.

**Methods:**

The acute episode have been treated conservatively with nasogastric decompression, intravenous replacement of fluids and electrolytes, antibiotics chemoprophylaxis and low molecular weight heparin. His overall physical status was unremarkable for obesity, diabetes, hypertension, allergies, previous operations and a former endoscopic evaluation conducted in the recent past, which had ruled out malignant neoplastic disease.

**Results:**

A course of per os prucalopride have been instituted, which showed preliminary promising results in restoring proper bowel movements, without any serious side effect and without the need to discontinue his course with antipsychotics. Prucalopride is a 5 HT4 agonist which selectively binds to the receptors of the intestine, resulting in muscular contractions as well as clorium secretion from the mucosa promoting an osmotic defecation.The substance has been extensively use in the treatment of irritable bowel disease of the chronic constipation type.

**Conclusions:**

We suggest the more systematic use of this agent in this group of patients after proper endoscopic evaluation and restoration of all secondary causes of constipation.

**Disclosure:**

No significant relationships.

